# Stability Studies of Magnetite Nanoparticles in Environmental Solutions

**DOI:** 10.3390/ma14175069

**Published:** 2021-09-04

**Authors:** Urszula Klekotka, Elżbieta Zambrzycka-Szelewa, Dariusz Satuła, Beata Kalska-Szostko

**Affiliations:** 1 Faculty of Chemistry, University of Bialystok, Ciołkowskiego 1K, 15-245 Białystok, Poland; u.klekotka@uwb.edu.pl (U.K.); elazamb@uwb.edu.pl (E.Z.-S.); 2 Faculty of Physics, University of Bialystok, Ciołkowskiego 1L, 15-245 Bialystok, Poland; d.satula@uwb.edu.pl

**Keywords:** nanoparticles, particles stability, environmental pollution, hazard

## Abstract

In the presented paper, studies of magnetite nanoparticle stability in selected environmental solutions are reported. The durability tests were performed in four types of liquids: treated and untreated wastewater, river water, and commercial milk (0.5% fat). Nanoparticles before and after deposition in the testing conditions were measured by transmission electron microscopy, X-ray diffraction, infrared spectroscopy, and Mössbauer spectroscopy. The amount of Fe atoms transferred into the solutions was estimated on the basis of flame atomic absorption spectroscopy. The analysis of the obtained results shows good stability of the tested nanoparticles in all water solutions. They do not change their structure or magnetic properties significantly, which makes them a good candidate to be used as, for example, detectors of specific compounds or heavy metals. On the other hand, studies show that particles are stable in environmental conditions for a long period of time in an unchanged form, which can cause their accumulation; therefore, they may be hazardous to living organisms.

## 1. Introduction

One of the main reasons why magnetite nanoparticles have become a popular subject for so many studies is their universality, low toxicity to living organisms, and relatively high biodegradability [1,2,3,4]. The fact that iron oxides are easily biodegradable significantly reduces the risk of dangerous environmental pollution caused by nanoparticles after the fulfillment of their function. The drawback is that the solubility of magnetite is rather low in most solvents [5,6], which provides new advantages and allows them to be used in medicine or environmental protection [7,8,9]. To add to their primary functions new properties, simple single-phase nanoparticles can be modified layer-wise to obtain more advanced core–shell structures [10,11,12]. Such morphology helps to prevent their degradation and therefore its pollution degree, or improving their dissolution process to avoid the toxicity of the waste. 

The core–shell nanoparticles, the core of which is made of magnetite and the shell of which is prepared from noble metals [13], polymers [14,15,16], or functional organic layers [17,18], possess unique magnetic properties accompanied by additional features such as optical responsive layers or bio function or specific reactivity, which is achieved due to the presence of hybrid particle core–shell morphology [19,20,21,22].

Knowledge of the extraordinary structural and magnetic properties of these materials permits the performance of modifications on the objects obtained by superficial functionalization with compounds containing free functional groups such as, for example, amine, carboxylic, phosphonic, or thiolated [23,24,25]. The presence of such groups on the surface allows, for example, the capturing of specific compounds (heavy metals, derivatives of medicaments, pesticides, etc.) in a relatively well-controlled way [26,27,28]. The resultant composite heterostructures can be successfully extracted from a solution employing the simplest sources of an external magnetic field (strong enough permanent magnets). Establishing a nanosized functionalized composite material is an innovative solution and therefore can significantly contribute to the purification of surface water, groundwater, and everyday products from defined pollutants [26,29,30]. For that reason, the studies described in the following paper have immense application potential. Materials and their properties presented here are likely to solve plenty of current problems such as, for instance, environmental protection, waste reduction, resource circulation, and the accumulation of pollutants. One may suggest undemanding separation methods for toxic compounds or/and heavy metals from surface water, groundwater, wastewater or everyday products with the aid of specially modified nanoparticles that can act as “detectors” of defined compounds through its adsorption on the surface, which allows their subsequent separation from the solutions by using an external magnetic field sources.

The market for high technology that enables the rapid and effortless purification of water is immense around the world. Apart from that, this demand is increasing due to the development of many factories, which, in most cases, consume high amounts of available drinking water or pollute it heavily. Consequently, the suggested nanocomposites may be found in both industrial water treatment for small and big cities and households.

The presented paper is a continuation of the durability studies on the nanoparticles that have been modified in different ways and maintained in particular model solutions [31,32,33]. The reason for these studies is based on the idea of developing a new class of filters in which purposely modified magnetite nanoparticles can be used as well as to show the potential of hazards related to the long-lasting migration of nanoobjects that have been introduced into the real environment.

## 2. Experimental

### 2.1. Materials and Apparatus

The nanoparticles used for the stability tests in environmental solutions were made from the following chemicals, which were purchased from: (a) Sigma-Aldrich, Darmstad, Germany: Fe(acac)_3_, 1,2-hexadecanediol, phenyl ether, and tetrabutylammonium hydroxide (TBAOH); (b) Avantor (Gliwice, Poland): FeCl_3_·6H_2_O, FeCl_2_·4H_2_O, HCl, NaOH, citric acid, acetone, NH_3_, and oleic acid; and (c) Fluka (Sigma-Aldrich, Darmstad, Germany): 98% tetraethoxysilane (TEOS), tetramethylammonium hydroxide (TMAOH), 1-octadecanol, and oleyl amine.

Cleaning, separation, and size limitation of the nanoparticles were performed with the use of a sonication bath and a permanent magnet.

All of the examined nanoparticles were measured to check their initial and final crystalline structure using X-ray diffraction (XRD) (Agilent Technologies SuperNova diffractometer (Yarnton, Oxfordshire, UK) with a Mo micro-focused source (K_α2_ = 0.713067)). Transmission electron microscopy (TEM) measurements were conducted with a FEI Tecnai G2 X-TWIN 200kV microscope (Thermo Fisher Scientific, Hilsboro, USA), where the nanoparticles were placed by means of drop-casting onto the carbon-covered 400 mesh Cu grid after dissolution. Infrared spectra (IR) were collected in a spectral range between 400–4000 cm^−1^ and was performed with a Nicolet 6700 spectrometer (Thermo Fisher Scientific, Hilsboro, USA) working in a reflecting mode. Mössbauer spectra were obtained with the spectrometer working in a constant acceleration mode with a ^57^CoCr radioactive source. The velocity scale was calibrated using α-Fe standard foil at room temperature. The iron concentration in the tested environmental solutions was measured by means of flame atomic absorption spectrometry (FAAS) (ICE 3500 with 1.2 L/min gas flow, burner height of 13.4 mm, and air-acetylene flame). For every measurement volume of 4 cm^3^ of liquid was used. Absorbance signals were the mean based on three repetitive measurements. The D2 (deuterium lamp) was applied for background correction. The quantitative determination of Fe was conducted using the external calibration curve technique.

### 2.2. Preparation of Nanoparticles

The magnetic nanoparticles used in the present studies were obtained from three different synthesis methods. In the first (A) set, magnetite particles were obtained according to the Massart synthesis procedure [34]. In the second type (B), the nanoparticle core was modified by a silica shell. The main steps used for the synthesis were adopted from the Wagner method [35]. In the last series (C), the procedure first published by Sun was used [36]. A more precise description of the three types of nanoparticles studied in this work is as follows:

(A) In this case (Fe_3_O_4_ NP’s), the so-called Massart synthesis was used. This method is based on the co-precipitation of Fe(III) and Fe(II) chlorides in ammonia aqueous solution at the temperature of 80 °C under an Ar atmosphere. In two flasks, a proper amount of FeCl_3_·6H_2_O and FeCl_2_·4H_2_O was dissolved in ammonia solution. In the next step, TBAOH was added to both flasks. The mixture with the Fe(II) chlorides was combined with the flask containing the Fe(III) chlorides, and the whole solution was heated to 80 °C.
(B) To obtain magnetite with a silica shell (SiO_2_@Fe_3_O_4_ NP’s), in a similar manner to the previous nanoparticles, two round-bottom flasks were used. However, in this case, in the first flask, distilled water was used, and in the second flask, a solution of distilled water with 37% HCl was deoxygenated. Then, FeCl_3_·6H_2_O was added to the first flask, and FeCl_2_·4H_2_O was added to the second flask. Both solutions were heated to 50 °C, and at the same time, vigorous stirring in an argon flow was continued. The solution from the second flask was slowly combined with the solution from the first flask, and after the addition of 1 M NaOH, the mixture was heated to 95 °C. The resultant mixture was still blended and deoxygenated for the next 30 min [37].
  When the resultant precipitate was cooled down, the excess citric acid solution of 0.01 M was added to the mixture. Then, a TMAOH solution was poured until the pH reached value 7. With such a solution, a deoxygenated mixture of ethanol, distilled water, and 25% ammonia was combined and mixed for about 10 min. After that, a TEOS solution was slowly added and was left for 24 h at RT on the stirrer to start a reaction.
(C) In the last synthesis (Fe_3_O_4_@Fe_3_O_4_ NP’s), Fe(acac)_3_ salt was used to obtain seed particles. The mixture of 8 mmol Fe(acac)_3_, 1,2-hexadecanediol, phenyl ether, oleic acid, and oleyl amine was stirred and heated to 260 °C for 30 min with continuous argon flow. When the solution had cooled to room temperature, Fe(acac)_3,_ 1-octadecanol, oleic acid, and oleyl amine were added. After that, the whole mixture was heated once again, this time to 230 °C for 30 min [36,38].

Particles from every synthesis were washed 3 times with deoxygenated acetone in the presence of a permanent magnet and were dried in a vacuum evaporator to obtain a powder form.

Nanoparticles from either synthesis were flooded with solutions of treated and untreated wastewaters, river water, and 0.5% fat milk. The pH values of the tested solutions are as follows: treated wastewater (7.9), untreated wastewater (7.7), river water (7.6), milk (6.6). The influence of wetting the nanoparticles in the selected environmental solutions and their possible changes after 1, 2, and 3 weeks were measured. After every week, a set of the solutions was removed with the help of a permanent magnet, and the nanoparticles were dried at RT (room temperature) for a minimum of 24 h before further characterization.

To reduce the amount of data in the paper, only the experimental results (TEM, XRD, FAAS, IR, and MS) after 3 weeks of wetting are presented. For weeks 1 and 2, the experimental duration the observed changes of the particles and solutions are in good correlation with the data shown here.

## 3. Results and Discussion

### 3.1. TEM Studies

**Figure 1:**
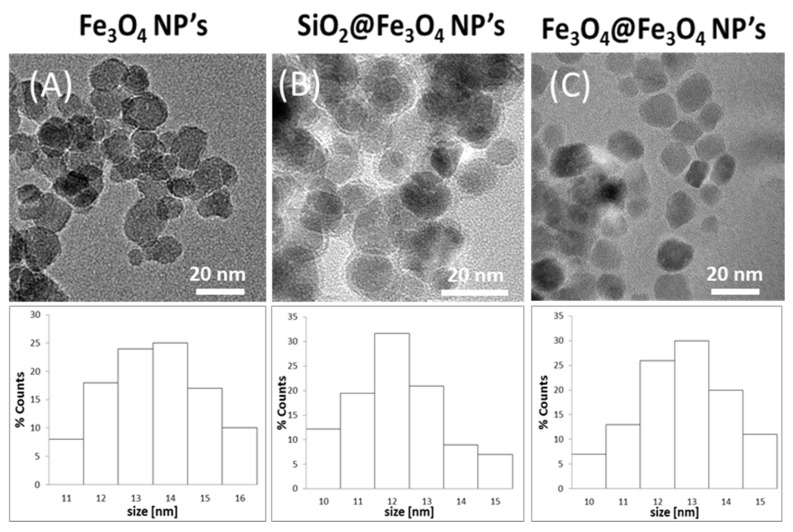
TEM images of magnetite nanoparticles from (**A**) iron chlorides; (**B**) with a silica shell; (**C**) from iron acetylacetonate and respective histograms for every type of tested nanoparticles.

The morphology of the magnetite nanopowders before wetting was imaged using transmission electron microscopy. The evaluated obtained results are shown in Figure 1 where they are depicted along with raw pictures and their respective histograms showing size distribution.

The presented TEM images indicate that the nanoparticles that have been selected for tests have a very similar shape and size, which was estimated to be 13 ± 2 nm for Fe_3_O_4_; 12 ± 1 nm for SiO_2_@Fe_3_O_4_; and around 12 ± 2 nm for Fe_3_O_4_@Fe_3_O_4_. The mean size of the nanoparticles is in good agreement with the data presented in the respective histograms. In the case of SiO_2_@Fe_3_O_4_, the SiO_2_ shell on the nanoparticles is clearly seen as a brighter shadow around particles.

### 3.2. Gravimetric and FAAS Results

To observe any mass changes related to the bathing procedure, the gravimetric studies were first performed. For that, the dry powders of the nanoparticles were weighed (around 100 mg) before being in the solutions. After 3 weeks of test duration, solid-fraction nanoparticles were separated from the solution with the assistance of the permanent magnet, dried in the air with argon flow, and then weighed again (for details see Table 1).

After 3 weeks of being in contact with the magnetite nanoparticles, the residual liquid was analyzed using AAS spectroscopy to observe the possible presence of Fe ions related to the core dissolution.

In Table 1, the results of the gravimetric measurement before and after 3 weeks of wetting time correlated with the FAAS results in respect to the various solutions are collected.

The gravimetric results of the presented Fe_3_O_4_ nanoparticles kept in the experimental environment increased from 15% up to almost 50%. A mass gain suggests an adsorption of the third particles or the organic compounds from the solution onto the particle surface rather than the dissolution of the inorganic core. Such a scenario is in good agreement with the FAAS results. In most of the tested solutions, the iron concentration was below the limit of detection (6 µg /L) [39].

On contrary, for the SiO_2_@Fe_3_O_4_ nanoparticles, the mass decreased in a range from 15% to about 19%. However, no traces of any Fe atoms in any of the tested solutions were revealed. This leads to the conclusion that the observed mass loss is caused by the degradation of the incomplete and loosely bounded silica shell or by the rinsing of some of the residual chemicals from the tested particles that have been transferred onto the solid particle surface from the synthesis environment. The comparison with the gravimetric results for the nanoparticles wetted in artificial conditions, among others, in distilled water, which was published in previous studies (their mass loss was observed to be about 3%) [33], shows that the silica shell “degrades” more in environmental solutions.

For the last type tested material (Fe_3_O_4_@Fe_3_O_4_), the mass increase is observed after the wetting of the nanoparticles in the treated and untreated wastewater and milk. This growth is at a level of up to 14%, and the mass loss for the nanoparticles wetted in the river water reached 6%. Comparing the results obtained here (for untreated wastewater and river water) with nanoparticle wetting in model solutions with distilled water (mass loss 4%), it can be concluded that this kind of nanoparticle has similar stability in artificial model solutions to real conditions [31].

The FAAS results show a negligible (zero with the experimental accuracy) amount of Fe atoms in the solutions, and these are comparable with all of the described cases. Therefore, no significant changes to the cores of the particles that have been induced by the used solutions can be seen. These results show that each kind of nanoparticle, Fe_3_O_4_, SiO_2_@Fe_3_O_4_, and Fe_3_O_4_@Fe_3_O_4,_ presents strong durability in the tested environmental solutions. They remain stable and do not dissolve under the present conditions. Nevertheless, weight modulations suggest a modification of the nanoparticles at the surface due to the composition of the media. The obtained results have a main significant drawback, i.e., the fact that all nanoparticles deposited in waste remain unchanged and can survive for a long period of time in any kind of waste in the environment. According to the reference results, only an acidic environment can influence the magnetite core more drastically. This causes a reduction in the size and degradation (further oxidation) [31,33].

### 3.3. X-ray Studies

**Figure 2:**
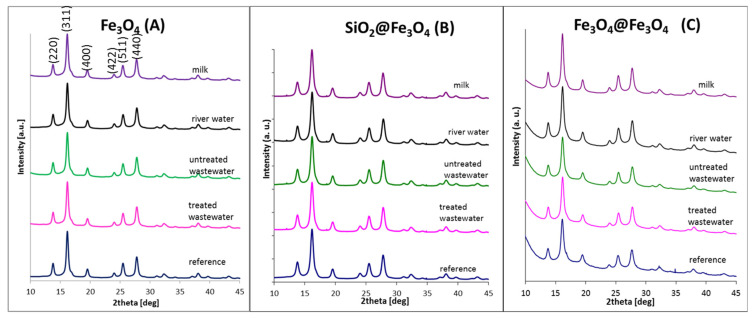
X-ray diffraction patterns of tested nanoparticles in various environmental solutions: (**A**) Fe_3_O_4_; (**B**) SiO2@Fe_3_O_4;_ (**C**) Fe_3_O_4_@Fe_3_O_4_.

Wetted nanoparticles were examined by means of X-ray diffraction to analyze any possible changes in the crystalline structure of the inorganic core as a consequence of the influence of the solution. The results obtained in the series are depicted in Figure 2.

XRD results prove previous conclusions and fulfill our expectations since the presented series of tested nanoparticles do not show any significant changes in the crystalline structure suggesting an oxidation process, which is an indication of nanoparticle destruction in the presented conditions. These results are also in accordance with the FAAS data. In every set of diffraction patterns, a well-defined magnetite crystalline structure with indexes hkl ascribed as (220), (311), (400), (422), (511), and (440) [40,41] are seen. These results are also in good correlation with our previous studies, where selected nanoparticle degradation was tested in other arbitrarily chosen artificial solutions [31,33].

To determine the average grain size of the tested nanoparticles, the Williamson–Hall equation (1) [42] was used.

(1) $$\beta cos\theta =\left(\frac{0.9\lambda }{D}\right)+\left(4\varepsilon sin\theta \right)$$

where *D*–grain size [Å], *λ*–wavelength (for Mo source is 0.7136 Å), *β*–full width at half maximum intensity of the (311) peak [rad], *ε*–strain, and *θ*–diffraction angle (311) line [rad].

Based on Equation (1), the calculated crystal parameters for each type of particle were compiled in series in Table 2.

The results obtained from the above equation show that both tested types of nanoparticles have almost the same grain size, which is in good agreement with TEM imaging. Additionally, the strain and lattice constant are similar in every case. The calculated lattice parameters are in good agreement with theoretical values for magnetite (8.39 Å) [5] and for maghemite (8.33 Å) [43], which are indistinguishable by XRD. A minor modification of the structure’s surface that has been influenced by particle treatment cannot be observed by XRD, and average grain size, which is within the error bar, is unchanged regardless of treatment conditions. Any deviations in XRD patterns not only indicate particle size variations but also the particle’s crystallinity, structural defects, chemical variations, surface effects, etc., which is the case here.

### 3.4. IR Studies

**Figure 3:**
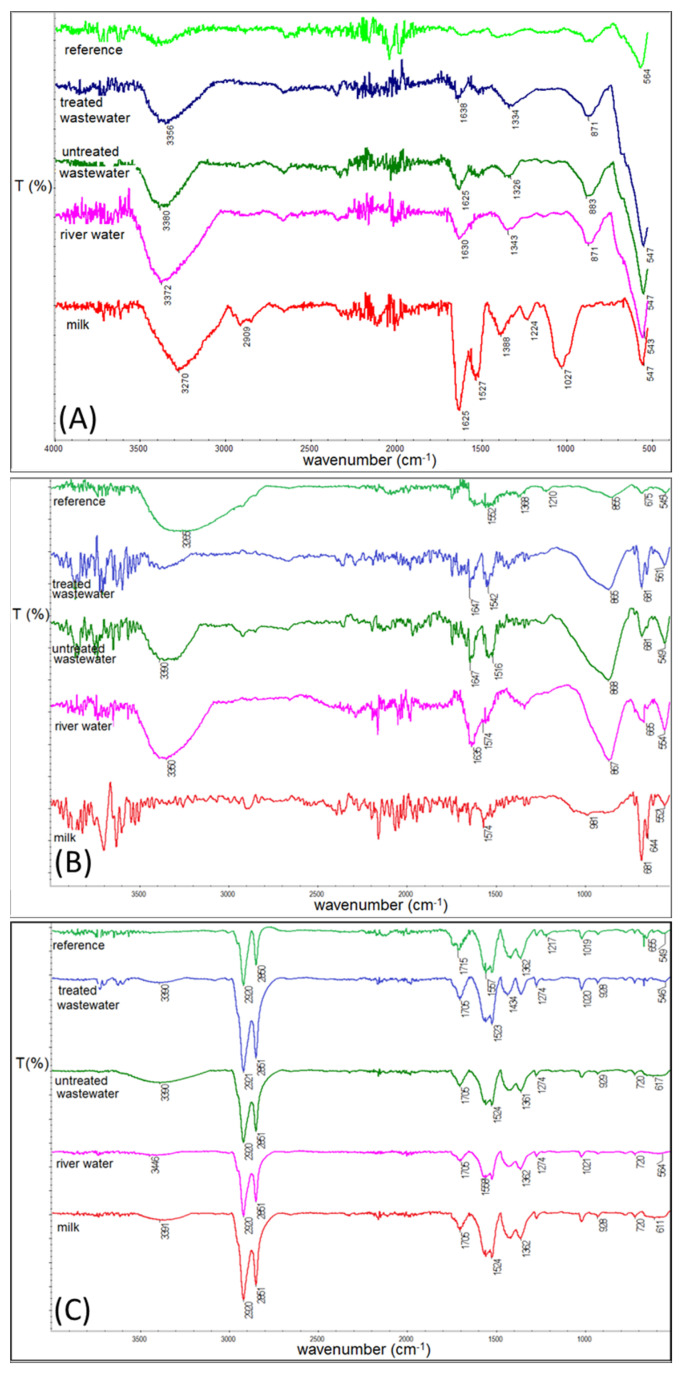
IR spectra of both kinds of tested nanoparticles in various environmental solutions. (**A**) Fe_3_O_4_; (**B**) SiO_2_@Fe_3_O_4_ nanoparticles; (**C**) Fe_3_O_4_@Fe_3_O_4_ nanoparticles.

After each wetting period, magnetite nanoparticles were tested by means of IR spectroscopy to determine changes in the nanoparticle surface caused by the chemical composition of the tested solutions. Since the most pronounced changes in the IR spectra are seen after 3 weeks of wetting, such particle spectra were chosen for presentation. After 1 and 2 weeks, the spectra have the same features in each solution. The spectra of every kind of nanoparticle wetted for 3 weeks are collected in Figure 3. 

IR spectra of Fe_3_O_4_ nanoparticles (Figure 3A) after wetting in untreated wastewater, treated wastewater, and river water do not show any significant changes in comparison to the reference. Only bands identified as Fe-O bonds in goethite (870 cm^−1^) [43] and maghemite (around 650 cm^−1^) [44] become more intensive. On the contrary, the IR spectrum of the nanoparticles wetted in milk shows a strong additional signal (1030 cm^−1^), which may be related to fat and proteins residues [45]. The comparison of the reference spectra in Figure 3B with the others shows that nanoparticles tend to oxidize into maghemite. In this case, this phenomenon is the most observable in milk, where an intense band at 681–651 cm^−1^ appears [44] and dominates a band that is typical for magnetite (550 cm^−1^) [46]. FTIR spectra also prove the modification of the nanoparticle surface with silica. Here, Si-O and Si-CH_3_ are observed at 808–867 cm^−1^ [45]. Again in milk, the band in the range of 1000 cm^−1^–1100 cm^−1^ becomes more pronounced. Spectra presented in frame (C) are the least modified by the wetting procedure. All other bands observed in the spectra are typical for the respective type of synthesis and were described elsewhere [31]. The analysis of the IR studies allows for the conclusion that the examined nanoparticles have relatively good stability in the tested solutions, whereas a variable environment has a very limited degradation effect on the magnetite core. Weak IR signals shift, and no traces of additional crystal phases in the XRD data suggest only slow transformation taking place between Fe oxides.

### 3.5. Mössbauer Studies

**Figure 4:**
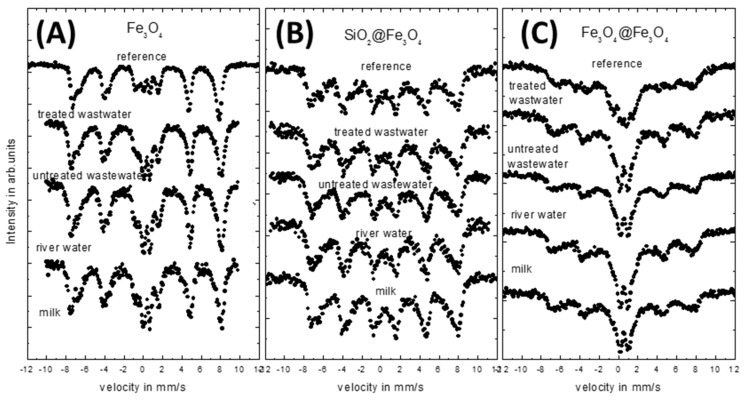
RT Mössbauer spectra of all tested types of nanoparticles wetted in various environmental solutions. (**A**) Fe_3_O_4_; (**B**) SiO_2_@Fe_3_O_4_ nanoparticles; (**C**) Fe_3_O_4_@Fe_3_O_4_ nanoparticles.

Collected RT Mössbauer spectra of the tested nanoparticles after 3 weeks wettings are depicted in Figure 4 in respective series.

As it can be seen from Figure 4, the shape of the RT Mössbauer spectra of the reference samples obtained by three different synthesis procedures is clearly different even for the particles that are similar in size. This is related to the crystal growth condition and surface modification related to routine of synthesis [38]. The spectrum of the Fe_3_O_4_ serie (A) sample is the superposition of two relatively sharp sextets and a small doublet in the middle of the spectrum. In the case of SiO_2_@Fe_3_O_4_ (B), only broad sextets are seen. In the case of Fe_3_O_4_@Fe_3_O_4_ (C), the spectrum is a superposition of a broad sextet and a very intensive doublet. This observation can be due to few reasons. First of all, each synthesis leads to a slightly different morphology of the particles, which depends on the average diameter, shape as well as size distribution of the obtained particles. In our synthesis procedures, the particle size changed (as estimated from TEM) from about 13 ± 2 nm for Fe_3_O_4_ to 12 ± 2 nm for both SiO_2_@Fe_3_O_4_ and Fe_3_O_4_@Fe_3_O_4_. The second reason is connected with the crystallinity of the nanoparticles obtained by different methods. In the case of the synthesis of the chlorides, one can expect well-defined growth, and therefore, monocrystal particles are expected [47]. Different crystallinity of the inorganic core can be obtained during Fe_3_O_4_@Fe_3_O_4_ synthesis from iron(III) acetylacetonate due to layer-by-layer growth. In the case of nanoparticles prepared with a silica shell, the interaction between iron atoms and the SiO_2_ shell, or the oxidation protection of the Fe_3_O_4_ core by the SiO_2_ shell should be also taken into account. All of this influences particles appearing and their magnetic properties, especially the ferrimagnetic–superparamagnetic transition and the surface activity of the studied nanoparticles [48].

The quantitative analysis of the reference spectra measured for Fe_3_O_4_ nanoparticles (Figure 4A) suggests that the nanoparticles are below superparamagnetic blocking temperature; therefore, sharp sextets and a small doublet are seen here (its relative intensity is much less than 50% of the spectra). The middle panel (Figure 4B) reference spectrum presents the measurements of the SiO_2_@Fe_3_O_4_ particles, where each sextet line is broadened by the presence of smaller objects and a complicated morphology caused by the influence of the SiO_2_ shell on the Fe_3_O_4_ core. Here the characteristic surface doublet in the central part of the spectrum is not visible. Therefore, these spectra also show that the particles are well below the superparamagnetic fluctuation regime. In the last case of the measured reference Fe_3_O_4_@Fe_3_O_4_ nanoparticles (Figure 4C), the obtained spectrum is a superposition of a very broad sextet and doublet with relative intensity above 50% of the total area. This means that the particles at room temperature are in the superparamagnetic state, and the blocking temperature is close to RT.

The shape of the Mössbauer spectra of all of the studied nanoparticles treated by different environmental solutions show the rather limited influence on their magnetic properties (Figure 4). In the case of SiO_2_@Fe_3_O_4_, there are no visible changes in the spectra for all of the used solutions. This means that the SiO_2_ shell protects the influence of a solution on the magnetite core. However, for Fe_3_O_4_ and Fe_3_O_4_@Fe_3_O_4_, slight changes of the nanoparticles reflected in the relative intensities of the doublet and sextet are seen. For both series of the tested samples, the intensity of the doublet increases in comparison to the reference samples. The increase in the intensity of the doublet is almost the same for all of the solutions. Such behavior suggests changes in the morphology and composition of the studied nanoparticles, at least at the surface region (absorption of the organic substance on the surface). This leads to an increase of nanoparticles separation and a consequently weakening of the dipole–dipole interaction, or a possible progressive oxidation process of the Fe_3_O_4_ core. The presented scenario is in line with other presented data (FAAS and IR, for example).

## 4. Conclusions

Particles treated with environmental solutions are more stable in comparison to the ones tested in model solutions [31]. Low dilution in wastewater suggests that environmental solutions do not show enough acidic properties, as is the case of, for example, pure acetic acid solution. The biggest mass loss was observed for the particles with the SiO_2_ shell, but at the same time, no presence of Fe in the solution was marked. This suggests partial (degradation) dissolution of the SiO_2_ shell. Despite that, the Fe ions were not transferred into the solutions neither in the case of the naked magnetite nor in the case of the magnetite covered in SiO_2._ The obtained results confirm the protective role of the SiO_2_ layer. Its presence prevents any changes in the core’s magnetic properties. In both pure magnetite series, reorganization of the structure (without Fe leaching) can be detected.

The presented results can be discussed and summarized in two ways. First, the good stability of nanoparticles means that they can be used with success as, for example, detectors of specific compounds or heavy metals [26,49] Their applications can be realized in a wide range of solutions. Due to the fact that the particles do not dissolve easily, their application possibilities increase. Second, the fact that nanoparticles do not dissolve in environmental solutions is a negative property for environmental protection because it means that they can accumulate/agglomerate in large amounts in many forms and can cause a significant pollution hazard over time.

## Figures and Tables

**Table 1:** A simultaneous presentation of mass changes of the tested nanoparticles before and after 3 weeks wetting, and the Fe amount in the different solutions (LOD-limit of detection).

	Fe_3_O_4_		SiO_2_@Fe_3_O_4_		Fe_3_O_4_@Fe_3_O_4_	
	Mass Change *±* 0.5 [mg]	FAAS <LOD	Mass Change ± 0.5 [mg]	FAAS <LOD	Mass Change ± 0.5 [mg]	FAAS <LOD
treated wastewater	14.9	0.00	−18.0	0.00	14.3	0.03
untreated wastewater	20.3	0.00	−18.9	0.00	3.2	0.00
river water	14.9	0.00	−17.6	0.00	−5.9	0.02
milk	51.9	0.05	−14.7	0.05	13.2	0.00

**Table 2:** Estimated average grain sizes, lattice parameters, and strain of wetted nanoparticles determined by X-ray diffraction patterns.

Type of Solution	Fe_3_O_4_ (A)			SiO_2_@Fe_3_O_4_ (B)			Fe_3_O_4_@Fe_3_O_4_ (C)		
	Grain Size ± 2 [nm]	Strain ± 0.5 [∙10^−3^]	Lattice Parameter ± 0.05 [Å]	Grain Size ± 2 [nm]	Strain ± 0.5 [∙10^−3^]	Lattice Parameter ± 0.05 [Å]	Grain Size ± 2 [nm]	Strain ± 0.5 [∙10^−3^]	Lattice Parameter ± 0.05 [Å]
reference	16	7.99	8.35	11	7.59	8.34	10	5.41	8.37
treated wastewater	17	7.90	8.36	10	6.11	8.33	10	6.38	8.36
untreated wastewater	17	7.76	8.36	10	5.69	8.33	10	6.56	8.36
river water	16	7.85	8.36	10	6.95	8.33	10	6.74	8.35
milk	16	7.87	8.37	10	6.35	8.34	11	6.82	8.35

## Data Availability

The raw data required to reproduce these findings cannot be shared at this time as the data also forms part of an ongoing study.
